# Rising total costs and mortality rates associated with admissions due to COPD exacerbations

**DOI:** 10.1186/s12931-016-0469-6

**Published:** 2016-11-14

**Authors:** Nicolas Molinari, Pascal Chanez, Nicolas Roche, Engi Ahmed, Isabelle Vachier, Arnaud Bourdin

**Affiliations:** 1 PhyMedExp, University of Montpellier, INSERM U1046, CNRS UMR 9214, Montpellier hospital, Montpellier, France; 2 Apard, Montpellier, France; 3 Département de Pneumologie et Addictologie, Hôpital Arnaud de Villeneuve, CHU Montpellier, Montpellier, France; 4 Service de Pneumologie et Soins Intensifs Respiratoires, Groupe Hospitalier Cochin, AP-HP, Université Paris Descartes, Paris, France; 5 INSERM CNRS U 1067 UMR7733, Université Aix-Marseille, Marseille, France; 6 Département des Maladies Respiratoires APHM, Laboratoire INSERM U1067, CNRS UMR 7333, Université Aix-Marseille, Marseille, France

**Keywords:** COPD exacerbation, COPD management, Hospitalizations, Admissions, Deaths

## Abstract

**Background:**

To examine trends in mortality, costs and in-hospital management and outcomes of severe COPD exacerbations admitted in France.

**Methods:**

Patients hospitalized from 2007 to 2012 with COPD exacerbation as the primary diagnosis were identified from the exhaustive French medico-administrative hospitalizations database records. Four groups of severe COPD exacerbations were defined: hospitalisation in a general ward (GW) without acute respiratory failure (ARF), GW with ARF, ICU without invasive mechanical ventilation (MV), and ICU with MV.

**Results:**

A 15.48 % increase in admissions from 113 276 in 2007 to 133 497 in 2012 was recorded. Age (+9.9 months), gender (−2.5 % of male) and length of stay (−0.29 day) slightly changed while the number of ICU admissions increased markedly (+41.78 %). In-hospital mortality rates increased (+8.06 %, *p* < .001) and followed seasonal variations peaking in winter. Total hospitalizations costs increased from 602 to 678 millions euros (+12.6 %). Pneumonia-related mortality increased (+37.2 %). A progressive replacement of chest X-ray by CT scan was observed (−41.3 % vs +31.7 %) while fewer spirometries (−13.7 %) and bronchoscopies (−22.6 %) were performed.

**Conclusion:**

The incidence of severe COPD exacerbations and the proportion of ICU-managed patients are still increasing in France. Rising total costs and mortality rates especially related to pneumonia advocate for rethinking COPD management plans.

**Trial registration:**

Not applicable.

**Electronic supplementary material:**

The online version of this article (doi:10.1186/s12931-016-0469-6) contains supplementary material, which is available to authorized users.

## Key messages

- What is the key question?

What are the precise trends in COPD admissions triggers and management and outcomes ?

- What is the bottom line?

The incidence of severe COPD exacerbations and the proportion of ICU-managed patients are increasing in parallel with total costs and mortality rates especially related to pneumonia.

- Why read on?

An exhaustive and consecutive national database analysis identified social determinants, cause of admissions and medical acts performed and identified great changes in the management which should be reappraised.

## Background

Chronic Obstructive Pulmonary Disease (COPD) is a frequent and devastating disease worldwide and will remain one of the major chronic diseases in the future [1]. Despite smoking bans in public places in most Western countries, COPD is still ascending and, by 2030, will remain one of the leading causes of death in Europe and westernized countries [2, 3]. Nowadays, as the general world population is aging, COPD patients have many comorbid conditions interacting with the respiratory disease and contributing to the difficulty of the long-term management [4, 5]. Noteworthy, COPD-related admissions are decreasing in Spain and in the US [6, 7]. Managing COPD is still challenging [8] despite critical achievements in the field of epidemiology, definition, mechanisms, and better understanding of the natural history of the disease. Positive effects of prevention and long-term management on survival and slowering of lung function are now likely achievable [9]. COPD affects the daily quality of life of most patients and represents a major burden for societies through both direct and indirect costs.

COPD exacerbations are major events in the natural history of the disease [10]. The “frequent exacerbator” phenotype is associated with a faster decline of lung function and health status, and poorer prognosis regarding the risks of hospitalization and death [11]. In addition, COPD exacerbation is a dreadful experience for patients, their relatives and the health care system [10, 12, 13]. Strategies aimed at improving the treatment and prevention of exacerbations remain far from being fully effective despite growing numbers of randomized controlled studies [14]. Current treatments do not target etiology-specific underlying mechanisms. Besides, some may be deleterious in the long term through increases in the risks of local (pneumonia) or systemic adverse events [15]. Costs related to COPD are mainly due to admissions for exacerbations [16]. All these considerations point to COPD exacerbations as a major challenge for the medical community and encourage research to better understand their burden, causes, outcomes and treatments. New opportunities to exhaustively address such questions arose in recent years since the quality control of registries dramatically improved in France with a policy based on three medical check-points (first, the doctor who codes, second, the dedicated doctor who checks and last, the payer who controls and who can impose fines in case of discordance).

In the present study, we report the results of a study investigating an exhaustive database of six consecutive years of severe exacerbations of COPD leading to hospitalizations in France. The data were obtained from the French admissions registry, which allows an exhaustive assessment of various trends. We aimed to understand whether features of COPD hospitalizations are changing in France with regard to incidence, severity, management, outcomes and costs.

## Methods

### Study design and population

This observational study used exhaustive standardized data from the national PMSI database (“Programme Médicalisé des Systèmes d’Information”), prospectively collected from 2007 to 2012. This dataset contains medico-economic information about admissions based on the International Classification of Diseases (ICD-10) classification from both public and private hospitals in France. It includes all data collected by healthcare providers before submission to the social welfare office. The study focuses on admissions for COPD from 2007 to 2012 (end of stay before January 1, 2013).

Hospitalization was defined by a stay of at least one night in a hospital. All patients with a permanent address in France were included. Patients with codes for chronic airway disorders other than COPD (e.g., asthma or bronchiectasis) were excluded in order to improve specificity. Only stays with COPD as a primary diagnosis were considered in order to avoid stays where COPD was quoted as a comorbidity. Sorting rules have already been described [17] and are recalled in the Additional file 1: Table SA1. When acute respiratory failure was used as a primary diagnosis, the case was considered only if COPD was confirmed as the related cause (i.e. quoted as associated diagnosis) of the acute respiratory failure.

Hospitalizations were categorized into four mutually exclusive categories; category 1: general ward without Acute Respiratory Failure (GW-no ARF); category 2: general ward with Acute Respiratory Failure, (GW-ARF); category 3: Intensive Care Unit without Mechanical Ventilation (ICU-No MV); and category 4: Intensive Care Unit with Mechanical Ventilation (ICU-MV). The data collected included age, gender, length of stay, vital status at discharge, month of discharge, patient home address and hospitalization costs for the French Health Care System. The top-five diagnoses quoted as related to the exacerbation were also analyzed. The most frequently reported medical acts relevant for managing COPD exacerbations were analyzed. Costs related to each stay was computed according to French’policy which takes inflation into account.

Socio-professional categories, CMU (Free Universal Health Care, corresponding to the lowest socio-economic categories, coverage rate, average household income) in each French ‘départment’ were obtained from the INSEE (Institut National de la Statistique et des Études Économiques).

### Statistical analysis

Hospitalization rates were standardized according to age and gender for each French ‘départment’. Descriptive data of quantitative variables were summarized as mean ± standard deviation and median with interquartile range. Unadjusted chi-square test was used for nominal or dichotomous variables, continuous variables were compared with the use of the unpaired *t*-test or the Mann–Whitney *U* test (according to the normality of the distribution, assessed with the Shapiro-Wilk test). The Mann-Kendall test was used to assess if there is a monotonic trend of the variable of interest over time. A linear regression was used to assess the association between hospitalization rates and economic variables. Due to large disparities of age-distribution between regions, age-standardized maps were built and these data were subsequently implemented in all statistical models where regions were used. Because the effectives are so large, virtually all of the *p* values are significant which makes them less useful. Subsequently, strong confidence in *p* > 0.05 was considered for non statistical significance. The statistical analysis was performed by the medical statistical department of the Montpellier University Hospital using SAS (version 9.3; SAS Institute; Cary, NC, USA) and R (version 3.1.1) statistical softwares.

## Results

Between 2007 and 2012, 733 585 hospital admissions corresponding to 600 662 COPD patients were recorded in France.

**Fig. 1:**
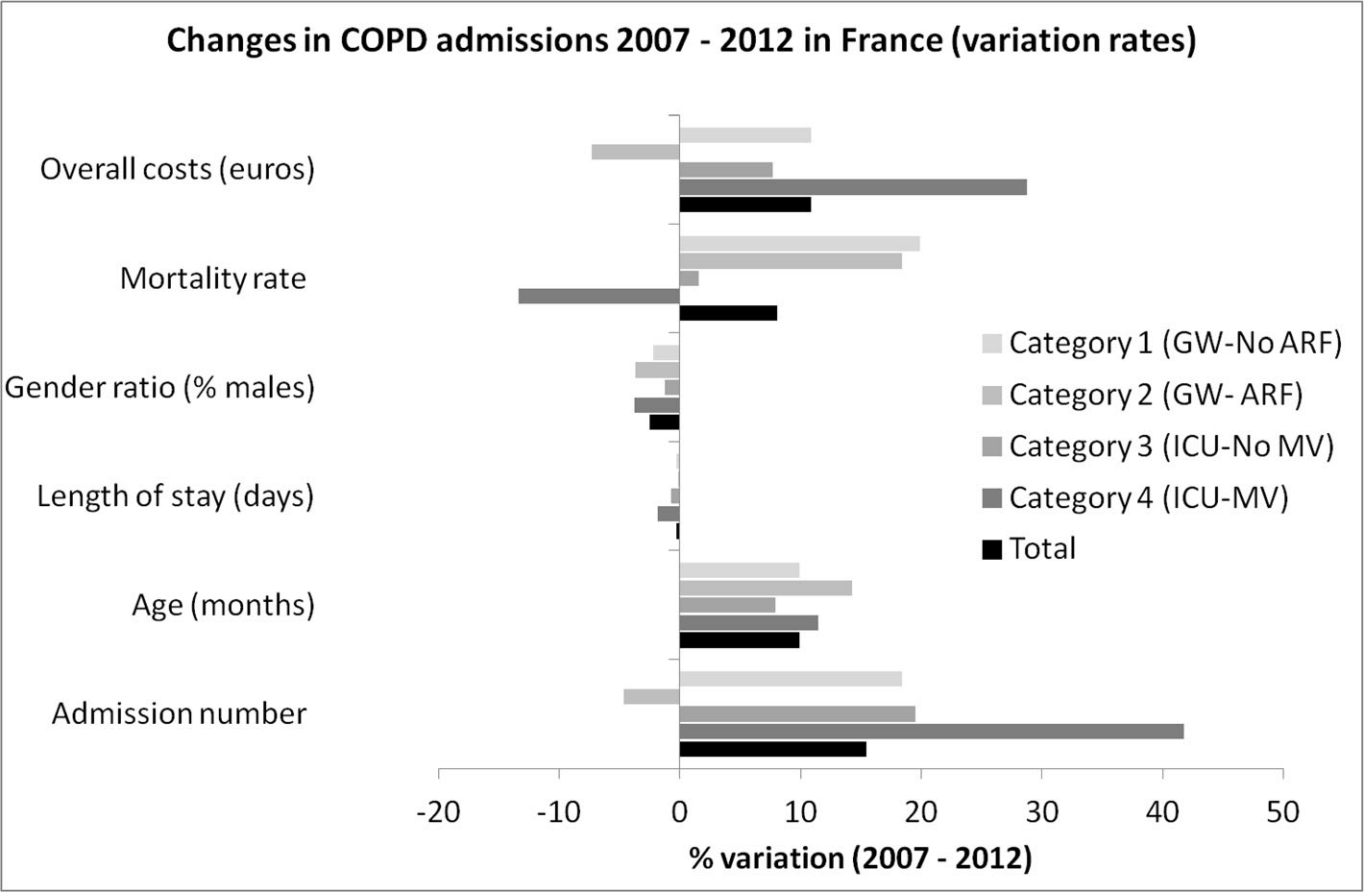
Trends in COPD admissions between 2007 and 2012 in France displayed by category of admission. Detailed numbers are provided in Table 1

**Fig. 2:**
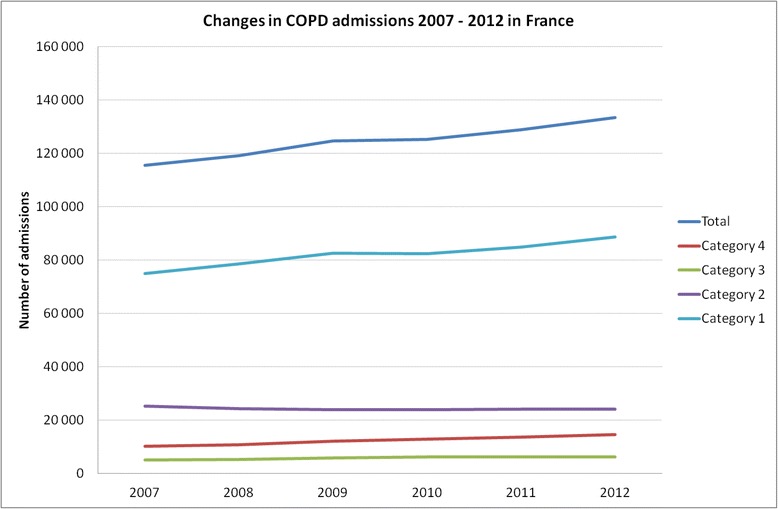
Trends in COPD admissions by category between 2007 and 2012 in France

Admission and demographic characteristics during the 6 years of interest and for each category of hospitalization are presented in Table 1 (given the large numbers, *p*-values are all significant and then not shown). Hospitalization categories 1, 2, 3 and 4 concerned respectively 64.9, 20.8, 4.6 and 9.7 % of admissions. Trends are displayed in Figs. 1 and 2. The number of hospitalizations increased by 18 % in 6 years (+24, −11, +23 and +48 % in catergories 1, 2, 3 and 4, respectively) while the overall French population increased by 2.59 % (63.601 in 2007 vs. 65.252 million people in 2012). Noteworthy, the proportion of patients corresponding to category 2 (GW ARF) decreased over time (−4.64 %, *p* < .001).

**Table 1:** Six-year Trends in hospitalizations for COPD exacerbation in France

Admission characteristics	Year	Total	Category 4 (ICU-MV)	Category 3 (ICU-No MV)	Category 2 (GW- ARF)	Category 1 (GW-No ARF)
Admissions (*N*, (%))	2007	115 600	10 239 (8.86)	5 155 (4.46)	25 227 (21.82)	74 979 (64.86)
	2008	119 105	10 811 (9.08)	5 274 (4.43)	24 382 (20.47)	78 638 (66.02)
	2009	124 669	12 061 (9.67)	5 912 (4.47)	24 008 (19.26)	82 688 (66.33)
	2010	125 331	12 876 (10.27)	6 184 (4.93)	23 916 (19.08)	82 355 (65.71)
	2011	128 847	13 611 (10.56)	6 130 (4.76)	24 192 (18.78)	84 914 (65.90)
	2012	133 497	14 517 (10.87)	6 160 (4.61)	24 056 (18.02)	88 764 (66.49)
Admission number change (% change and *p*-value)	2007/2012	+15.48 (<0.001)	+41.78 (<0.001)	+19.50 (0.065)	−4.64 (<0.001)	+18.39 (<0.001)
Age years, mean ± sd	2007	74.15 ± 12.05	70.49 ± 11.43	73.60 ± 11.48	74.31 ± 11.90	74.64 ± 12.13
	2008	74.25 ± 12.16	70.60 ± 11.75	73.36 ± 11.87	74.56 ± 11.96	74.72 ± 12.22
	2009	74.35 ± 12.20	70.58 ± 11.83	73.58 ± 11.76	74.77 ± 12.09	74.84 ± 12.22
	2010	74.45 ± 12.31	70.86 ± 11.79	74.02 ± 11.75	74.89 ± 12.20	74.92 ± 12.37
	2011	74.47 ± 12.34	70.50 ± 11.88	73.80 ± 11.91	74.99 ± 12.24	75.01 ± 12.36
	2012	74.97 ± 12.17	71.45 ± 11.57	74.26 ± 11.86	75.50 ± 12.07	75.46 ± 12.21
Age change (mo. change and *p*-value)	2007/2012	+9.9 (<0.001)	+11.5 (<0.001)	+7.9 (<0.001)	+14.3 (<0.001)	+9.9 (<0.001)
Length of stay years, mean ± sd	2007	10.89 ± 10.43	19.16 ± 19.01	13.38 ± 15.46	11.03 ± 8.98	9.53 ± 7.95
	2008	10.74 ± 10.39	18.85 ± 20.48	13.05 ± 11.17	10.93 ± 8.76	9.41 ± 7.89
	2009	10.76 ± 9.94	18.44 ± 18.02	12.95 ± 10.77	11.09 ± 9.48	9.39 ± 7.50
	2010	10.92 ± 10.11	18.26 ± 18.34	13.18 ± 11.24	11.08 ± 8.78	9.55 ± 7.78
	2011	10.74 ± 9.59	17.53 ± 16.51	12.69 ± 10.40	11.07 ± 8.93	9.41 ± 7.48
	2012	10.60 ± 9.82	17.32 ± 17.43	12.64 ± 10.39	10.92 ± 8.96	9.28 ± 7.53
Length of stay change (days change and *p*-value)	2007/2012	−0.29 (<0.001)	−1.8 (<0.001)	−0.74 (0.0355)	−0.11 (0.383)	−0.25 (<0.001)
Gender (% of Male)	2007	66.60	69.50	66.23	65.62	66.56
	2008	66.17	68.49	67.41	65.13	66.09
	2009	65.25	67.02	64.97	63.70	65.46
	2010	64.91	67.87	66.20	62.95	64.91
	2011	64.51	67.11	65.74	62.60	64.55
	2012	64.11	65.73	64.98	61.96	64.37
Gender ratio change (% change and *p*-value)	2007/2012	−2.50 (<0.001)	−3.77 (<0.001)	−1.25 (<0.001)	−3.66 (<0.001)	−2.19 (<0.001)
Deaths (*N*, (%))	2007	8 467 (7.32)	2 411 (23.55)	568 (11.02)	2 998 (11.88)	2 490 (3.32)
	2008	8 777 (7.37)	2 441 (22.58)	610 (11.57)	3 068 (12.58)	2 658 (3.38)
	2009	9 540 (7.52)	2 618 (21.71)	700 (11.84)	3 365 (14.02)	2 857 (3.46)
	2010	9 674 (7.72)	2 679 (20.81)	669 (10.81)	3 217 (13.45)	3 109 (3.78)
	2011	9 844 (7.64)	2 809 (20.64)	666 (10.86)	3 199 (13.22)	3 170 (3.73)
	2012	10 564 (7.91)	2 961 (20.40)	689 (11.19)	3 385 (14.07)	3 529 (3.98)
Mortality rate change (% change and *p*-value)	2007/2012	+8.06 (<0.001)	−13.38 (<0.001)	+1.54 (0.802)	+18.43 (<0.001)	+19.88 (<0.001)

*Abbreviation*: *NA* not applicable

Mean age at admission significantly increased from 74.15 ± 12.05 year to 74.97 ± 12.17 (+9.9 months). While men remained more numerous, the gender ratio trend showed a move toward equilibrium (−2.5 % of men, *p* < .001). Lengths of stays (9 ± 9 days) significantly decreased by 0.29 day.

Most characteristics were significantly different depending on the categories of hospitalization. Patients admitted in ICU requiring mechanical ventilation (category 4) were younger (*p* < .001), with longer length of stay (*p* < .001).

In-hospital mortality increased by 8.06 % (*p* < .001) depending on the categories of hospitalization (*p* < .001), the lowest mortality rates being recorded in category 1 (GW no ARF) and the highest in category 4 (ICU-MV). Over the 6 years, the mortality rate significantly decreased in ICU (category 4: from 23.6 to 20.4 %, *p* < .001) but increased for patients admitted in the ward (category 2 and 1: +18.4 and +19.9 % respectively, *p* < .001).

**Fig. 3:**
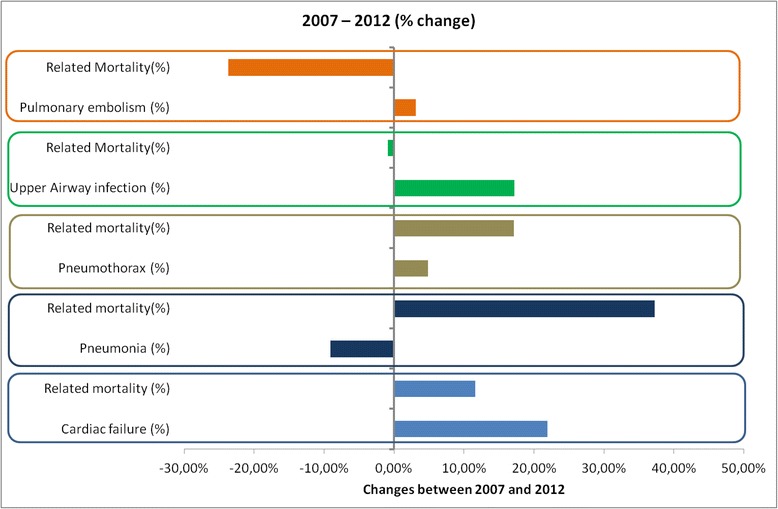
Trends in associated diagnosis with COPD admissions and related mortalities between 2007 and 2012. Detailed data are provided in Additional file 1: Table SA4

Overall, the costs per admission decreased in all four categories (Fig. 1) but the total costs of hospitalizations increased from 602 044 800 euros in 2007 to 667 885 491 euros in 2012 (+10.9 % vs. 2007, *p* < .001). The mean cost per admission was 3 667 ± 1 976 euros in 2012 depending on the hospitalization categories (from 9 243 for category 4 patients to 3 632 euros for category 1). The top cost for a single stay reached 337 000 euros. ICU contribution to overall costs remained nearly stable, with a transfer of 4 % from the category GW-ARF to ICU-MV (Additional file 1: Table SA3). The quoted acute conditions associated with exacerbations are described in Additional file 1: Table SA4. Basically, five final related diagnoses gathered 80 to 90 % of all stays. Upper airway infections overtook cardiac failure, pneumonias, pulmonary embolism and pneumothorax) (Fig. 3). A trend analysis identified a monotone rise in cardiac failure (*p* = .024) and upper airway infection (*p* = .009). The trend analysis identified a significant increase in the mortality related to pneumonia only (Mann Kendall *p*-value for trend *p* = .024).

**Fig. 4:**
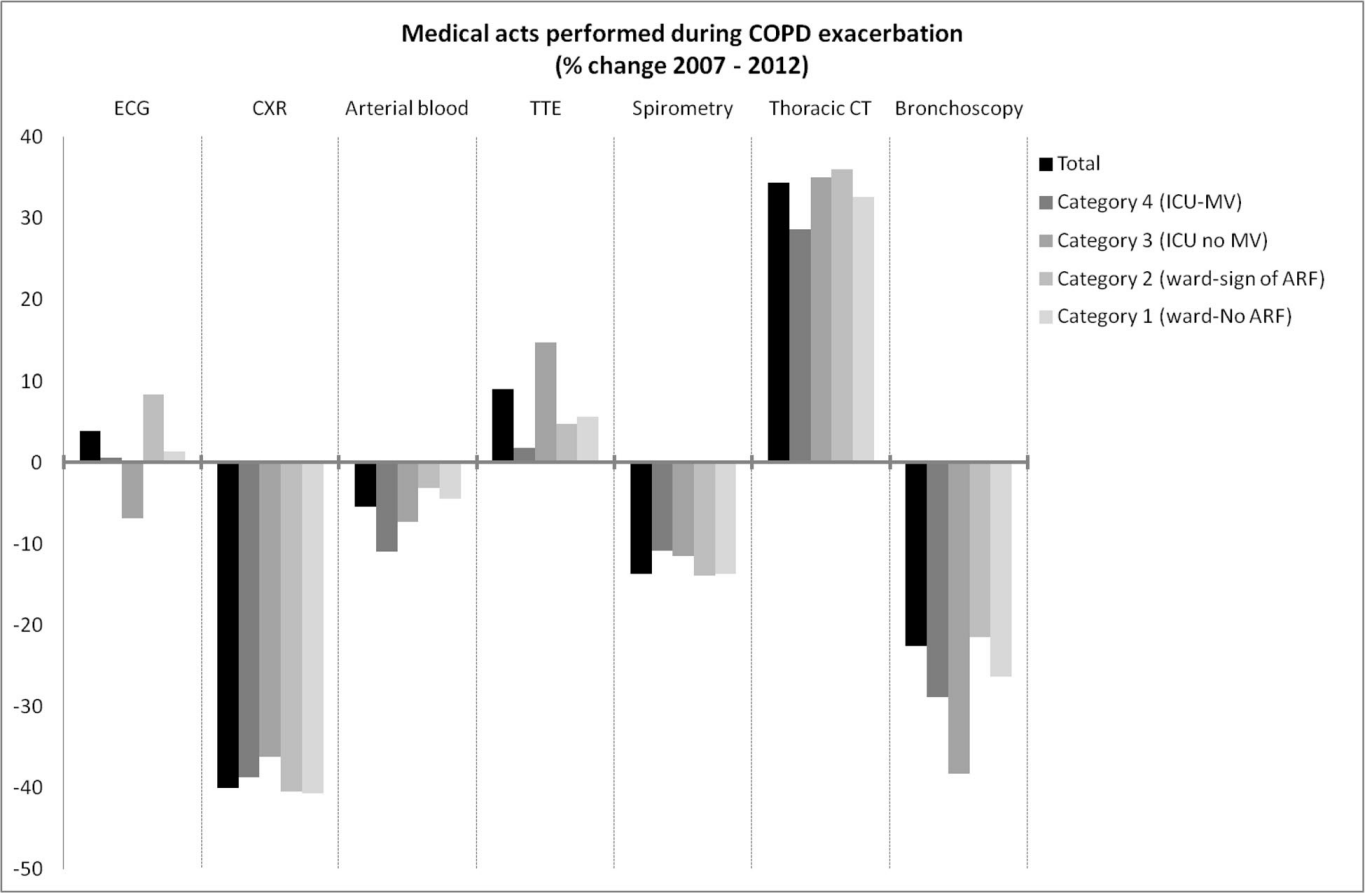
Trends in medical acts performed during COPD hospitalizations for exacerbations. (TTE = trans-thoracic echocardiography). Detailed data are provided in Additional file 1: Table SA5

Chest X-ray was the most frequent procedure performed during each stay (Additional file 1: Table SA5), followed by electrocardiogram and arterial blood gas analysis. There was a clear decrease in chest X-ray use (from 1.35 CXR to 0.81/stay, −40.1 %), while that of thoracic CT scan increased (+34.4 %). These trends were homogeneously recorded throughout the different categories of severity (Fig. 4). A monotone decrease in bronchoscopy was noticed (−22.6 %).

**Fig. 5:**
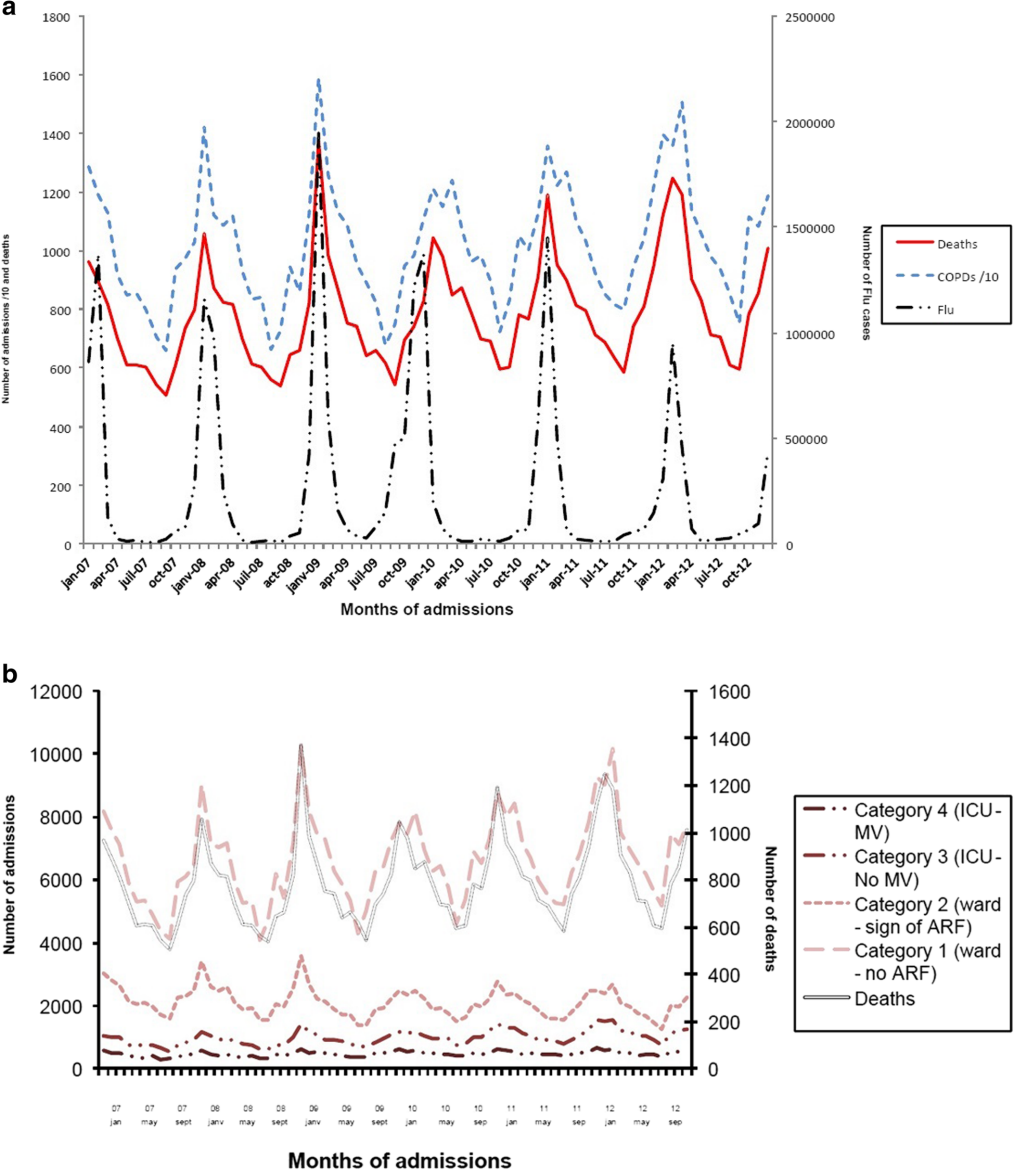
5**a** Seasonal changes in admissions (short dashed line) and deaths (solid line) due to COPD exacerbations in France from 2007 to 2012. Flu cases are reported on the same graph (long dashed line). 5**b**. Trends in COPD admissions and deaths in France among different categories

Seasonality of admissions and deaths are presented in Fig. 5a and b respectively. Data distributions were similar from 2007 to 2012. A non-uniform distribution of annual admissions (*p <* .001) and deaths (*p <* .001) were observed with a peak in incidence during the fall-winter season. This non-uniform distribution was also observed for admissions in each category. The seasonal peak was recorded at different dates each year, close to but not perfectly matching with FLU-peaks.

**Fig. 6:**
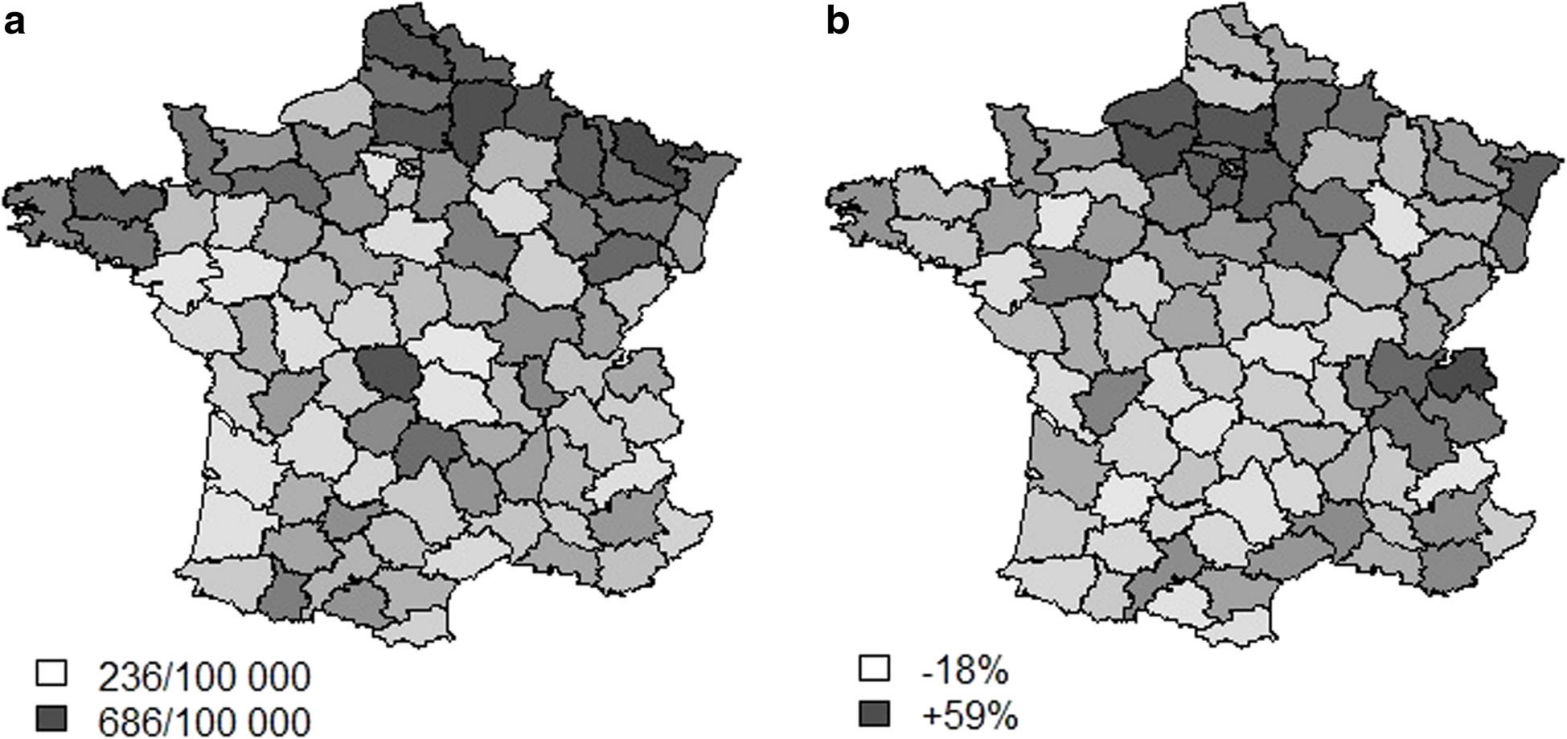
Geographical distribution of COPD hospitalizations (age-standardized) in France **a** incidence of admissions in 2012 (the darker, the greater the prevalence) and **b** regional trends in COPD hospitalizations between 2007 and 2012 (the darker, the greater the increase)

Spatial distribution of age-standardized hospitalization rates in 2012 is presented in Fig. 6a. Nearly three-fold regional heterogeneities were observed as admission rates ranged from 236 per year for 100 000 inhabitants to 686 per year for 100 000 inhabitants (*p* < .001). A north to south gradient was visually noticed. Regional variations from 2007 to 2012 are presented in Fig. 6b, displaying areas where COPD admissions increased the most. Regressions between age-standardized hospitalization rates and economic data of the inhabitants of the ‘départment’ were computerized. Lower age-standardized hospitalization rates in 2012 were associated with higher proportions of upper socio-professional categories (*p* = .004). There was no significant association with the CMU (Free Universal Health Care) coverage rate (*p*-value = 0.74). In contrast, when analyzing the variation of age-standardized hospitalization rates between 2007 and 2012 (areas were it increased the most), a negative correlation with average household income was found (*p* < .001, Adjusted R-squared = 0.304).

## Discussion

In this study, our ability to get exhaustive national consecutive data allowed to confidently identify three robust trends. First, incidence increases much faster than the population grows, especially regarding ICU-admitted patients. Patients are older, stays are shorter and women are more represented. Second, intra-hospital mortality also increases despite apparent improvement in the utilization of health care resources and the availability of funds specifically dedicated to this disease (as shown by an increasing of total dedicated expanse). Although costs were adapted, the overall bill increased along with the increasing incidence, with a greater part dedicated to ICU. Third, patterns of causes and in-hospital management of COPD followed deep mutations. In proportion, upper airway infection and cardiac failure increased while only mortality related to pneumonia increased. A progressive but massive replacement of chest X-rays by CT scans has been recorded while less spirometries and bronchoscopies were performed.

The main strengths of the present study rely on the exhaustiveness of data collection, largely resulting from enforced legal reporting obligations. Additionally, we have limited the classification bias by means of a strictly standardized patient identification algorithm which is strict and identical for all and has already been validated [17], with a view to sideline differential diagnoses and stays for which patients presenting COPD had not been admitted because of an exacerbation. Lastly, the fact that this study spans a period of 6 consecutive years makes its longitudinal quality a crucial strength potentially generalizable to most westernized countries.

Weaknesses inherently associated with such studies (lack of clinical data, missing values of pulmonary function tests, etc.) impose not to over-interpret any of those findings. Nonetheless, since 2007, a three-pronged quality control approach is applied to all dossiers (involving the coding doctor in charge of the patient, the dedicated doctor of the institution and the payer), which is the reason why we started our analysis at this period. We selected stays where COPD was quoted as the primary diagnosis only to limit the risk of coding bias especially in non specialized centres; subsequently our findings are quite specific of the disease but probably underestimated. Biases related to the epidemiological nature of the database are incontrovertible why trends assessed during six consecutive years likely represent the most meaningful part of the present study. Indeed, the fact that less spirometries were performed can diminish the rate of confirmation of COPD diagnoses, thus limiting the generalizability of the findings. This is highly true in ICU, but potentially also in non expert centres. Noteworthy, spirometry is rarely performed during exacerbation with the aim of confirming the diagnosis but rather to assess severity and/or as a discharge criteria [18].

The increasing incidence of COPD admissions observed here is different from what was reported in Spain and more generally in the Westernized countries [7, 19] in studies that used a very similar methodology. The definition of COPD may vary throughout these countries as a potential explanation [20]. A different level of severity restricted to France is another option; for instance the history of coal-mining as shown by our regional findings may contribute to these differences. The trends show that age and proportion of women increase with time. Besides, length of stay appears to shorten progressively.

The persistent increasing in-hospital mortality is surprising when considering the improvement in COPD overall management and regarding previous reports [21]. This is even more worrying since patients were in fact only slightly older (less than a year) and had no evidence of many coexisting diseases. This increase in mortality mainly occurred among ward-admitted patients – especially when clinical signs of acute respiratory failures were recorded. Even though ICU admissions increased, it seems that intermediate structures dedicated to non-invasive ventilation are still marginally available (what would correspond to category 3 of our classification), which could in part explain increasing in-ward mortality. It advocates for dedicated care from pulmonary divisions which should be expanded in the countries [18]. We need to discuss a decision algorithm to better adapt the destination ward to the patient needs.

The increases in mortality and incidence of hospitalizations are disappointing considering all efforts developed against COPD burden [1].

All available medications, vaccines, rehabilitation, comorbidities screening and management seemed not to sufficiently change the natural history of the disease.

We reported the five leading causes of exacerbation recorded as it gathered between 80 to nearly 90 % of stays, even though the exact definition of exacerbation and triggers is complex [22]. At most, we should consider our findings at risk of underestimation since this specificity is inherently related to a lower sensitivity. Among associated acute diseases, we observed a surprising increase in mortality related to pneumonia, leading to discuss several possible explanations. First, coding bias is unlikely since coding rules remained unchanged during the study period. Second, a change in the level of virulence of potential pathogens seems unlikely. Third, variations in Influenza or *Streptococcus pneumonia* vaccines effectiveness cannot be specifically addressed. A tendency to reduce the rate of vaccination in the general population and moreover in the elderly have been recorded in many countries as in France due to media-transmitted false fears of side effects, sometimes initiated by health care providers. Increasing use of ICS in COPD is again highly debatable regarding these observations. Antibiotics resistance is another possible explanation but currently available data are not supporting this hypothesis [23, 24]. Finally, the increasing use of CT-scan could have led to more diagnoses of pneumonia. Conversely, increasing incidence of upper airway infection remained associated with stable mortality rates. Cardiac failure was increasingly reported as a trigger of severe COPD exacerbations, which could partly relate to the increasing availability of NT-pro BNP assessment [19]. Of note, prevalence of pulmonary embolism in the context of COPD admission remained extremely stable and rare despite the increasing use of the CT scan– although details of CT acquisitions (i.e. suitable for detection of pulmonary embolism or not) were unavailable.

The geographic distribution of age-standardized hospitalization rates showed a very heterogeneous distribution, with more hospitalizations in the north of France, a historical mining region. Interestingly, the regions where the incidence increased the most are not the same and it can be hypothesized that occupational COPD is progressively vanishing. Data obtained from the monitoring of climatic and air pollution variables will probably be important to cross with the present epidemiological data in order to identify potential warnings usable at the individual level.

We found a relationship with socioeconomic data, a finding largely reported before. More unexpected, COPD admissions increased over the 6-year period of the study in the wealthiest areas, suggesting a change in the profile of COPD toward a richer, more feminine and less occupationally-exposed population.

Seasonality of admissions and deaths were reported before [25]. In the present data base, the peak of admission was disconnected from the FLU-peak some but not all years, and this represents a critical field of investigation.

This fact questions the immunization efficiency its use and acceptance within the COPD population with a real yearly variability.

Direct costs increased significantly (+10.9 %): although the established care package decreased over time, this evolution was overtaken by the increasing number of admissions. The relative contribution of each category to the overall costs was stable. The increasing number of ICU-admissions has been compensated by a decrease in the care package.

In the end, 34 % of the total costs came from the ICU contribution (which gained 4 % of the total expenditure) while accounting for 15.50 % of the total admissions.

The underlying motivation of this study was to identify areas of improvement. Interesting tools have been elaborated for more accurate identification of patients who should be admitted [26] while ambulatory management of COPD exacerbation at home was shown to be safe and efficient in carefully selected patients eligible to dedicated home management organizations [27].

## Conclusion

In conclusion, the trends reported here (notably the increasing incidence, costs and mortality of severe COPD exacerbations) represent a major warning for institutions, physicians and patients and should lead to extensively rethink COPD management plans and the allocation of dedicated health care resources in France.

## Additional file

Additional file 1: Table SA1. COPD hospitalizations and definitions of severity categories (sorting rules according to ICD-10) [17]. **Table SA2.** Leading cause associated with COPD admissions. **Table SA3.** Trends in admissions-related costs among different categories of severity. **Table SA4.** Cause of COPD admissions. Only five leading causes are mentioned. **Table SA5.** Top seven medical acts performed during hospitalizations for COPD exacerbations. 2007 and 2012 data and changes are presented. (DOCX 27 kb)
