# Supplementary material for: Rising total costs and mortality rates associated with admissions due to COPD exacerbations
Source: Respir Res. 2016 Nov 14;17:149. doi: 10.1186/s12931-016-0469-6 (PMC5109821; doi:10.1186/s12931-016-0469-6)
Supplement: Additional file 1: Table SA1. — COPD hospitalizations and definitions of severity categories (sorting rules according to ICD-10) [17]. Table SA2. Leading cause associated with COPD admissions. Table SA3. Trends in admissions-related costs among different categories of severity. Table SA4. Cause of COPD admissions. Only five leading causes are mentioned. Table SA5. Top seven medical acts performed during hospitalizations for COPD exacerbations. 2007 and 2012 data and changes are presented. (DOCX 27 kb) [file 12931_2016_469_MOESM1_ESM.docx]

**Rising total costs and mortality rates associated with admissions due to COPD exacerbations**

***A six-year exhaustive French national hospital database study***

Nicolas Molinari^1, 2^, Pascal Chanez^5, 6^, Nicolas Roche^4^, Engi Ahmed^1^, Isabelle Vachier^3^, Arnaud Bourdin^1, 2, 3^

ONLINE REPOSITORY

Supplement table legends

Table SA1: COPD hospitalizations and definitions of severity categories (sorting rules according to ICD-10)(19).

Table SA2: Leading cause associated with COPD admissions.

Table SA 3. Trends in admissions-related costs among different categories of severity.

Table SA 4. Cause of COPD admissions. Only five leading causes are mentioned.

Table SA 5. Top seven medical acts performed during hospitalizations for COPD exacerbations. 2007 and 2012 data and variation are presented.

Table SA1: COPD hospitalizations and definitions of severity categories (sorting rules according to ICD-10)(19).

|  | **Main diagnoses** | **Associates diagnoses** | **Remark** |
| --- | --- | --- | --- |
| Category 1: mild  (ward-No signs of acute respiratory failure) | J41 or J42 or J43 or J44 or J961+0 |  | Excluding: J45 and J46 and J47 and J80 and J95 and J98 and J960 and J961+1 and HDU and ICU |
|  | **or** |  |  |
|  | I26 or I50.0 or I270.0 or J13 or J14 or J18 or 20 or J40 or J93 or J100 or J110 or J120 or J121 or J122 or J123 or J128 or J150 or J151 or J152 or J153 or J154 or J155 or J156 or J157 or J158 or J159 or J160 or J168 or J170 or J851or U049 **plus associated diagnosis (see next column)** | **AND**  J41 or J42 or J43 or J44 or J961+0 | Excluding: J80 and J960 and HDU and ICU |
| Category 2: moderate  (ward sign of ARF) | Category 1 and (J80 or J960) | **AND**  J41 or J42 or J43 or J44 or J961+0 |  |
| Category 3: moderately severe  (ICU-No Mechanical Ventilation) | Category 2 and HDU |  |  |
| Category 4: severe  (ICU-Mechanical Ventilation) | Category 3 and ICU |  |  |

Table SA2: leading cause associated with COPD admissions

| Cause | ICD10 codes |
| --- | --- |
| Proximal airway infection | J100 or  J110 or  J200-J209 or  J40-J42 or  J44 |
| Cardiac failure | I270 or  I500 |
| Pulmonary embolism | I260 or  I269 |
| Pneumonia | J12-18 or  J85 |
| Pneumothorax | J93 |

Table SA 3. Trends in admissions-related costs among different categories of severity.

| Admission characteristics | **Year** | **Total** | **Category 4**  **(ICU-MV)** | **Category 3**  **(ICU-No MV)** | **Category 2**  **(GW- ARF)** | **Category 1**  **(GW-No ARF)** | **p-value (between categories)** |
| --- | --- | --- | --- | --- | --- | --- | --- |
|  |  |  |  |  |  |  |  |
| **Costs per admission per hospitalization**  **(euros, mean ± sd**  **median (Qrange)** | 2007 | 5208 ± 6227  4 617  (3 522-5 480) | 13 960 ± 15 080  9 671  (7109-15 540) | 7 381 ± 13 517  6 285  (5 096-7 949) | 5 250 ± 1 793  5 480  (5 448-5 480) | 3849 ± 1 563  4 568  (2 862-4 644) | <0.001 |
|  | 2008 | 5 033 ± 5 563  4472  (3409-5304) | 13 250 ± 14 897  9 360  (6 386-14 230) | 6 844 ± 5 488  6 103  (4 901-7 375) | 5 099 ± 1 678  5 304  (5 278-5 304) | 3 761 ± 1 533  4 425  (2 770-4 495) | <0.001 |
|  | 2009 | 5 063 ± 5 577  4 281  (2 865-5 574) | 13 750 ± 13 672  10 130  (6 877-15 830) | 6 925 ± 4 691  6 143  (4 864-8 100) | 5 243 ± 2 553  5 304  (4 705-6 337) | 3 611 ± 1 673  3 920  (2 316-4 495) | <0.001 |
|  | 2010 | 5 134 ± 5 712  4 045  (3 225-5 393) | 13 650 ± 13 796  9 894  (6 637-15 850 | 6 924 ± 4 469  6 083  (4 699-8 069) | 5 277 ±2 402  5 184  (4 194-6 348) | 3 627 ± 1 696  3 654  (2 287-4 553) | <0.001 |
|  | 2011 | 5 033 ± 5 252  5 033  (3 206-5 360) | 13 060 ± 12 233  9 567  (6 589-15 310) | 6 665 ± 3 811  5 998  (4 505-7 837) | 5 153 ± 2 339  5 152  (4 169-63 10) | 3 594 ± 1 636  3 632  (2 272-4 526) | <0.001 |
|  | 2012 | 5 003 ± 5 543  3 802  (3 211-5 138) | 12 680 ± 13 184  9 246  (6 409-14 480) | 6 653 ± 4 921  5 810  (4 456-7 714) | 5 104 ± 2 416  5 182  (4 177-6 310) | 3 606 ± 1 657  3 632  (2 262-4 535) | <0.001 |
| **Costs/stay variation**  **(euros change and *p*-value)** | 2007/2012 | -205  (<0.001) | -1280  (<0.001) | -728  (<0.001) | -146  (<0.001) | -243  (<0.001) | NA |
| **Variation in Overall costs**  **(% change and *p*-value)** | 2007/2012 | +10.9%  (<0.001) | +28.8%  (<0.001) | +7.7%  (<0.001) | -7.3%  (<0.001) | +10.9%  (<0.001) | <0.001 |
| **Relative contribution to overall costs in 2012**  **(Trends 2007-2012 between brackets)** | 2007/2012 | 100% | 28%  (+4%) | 6%  (unchanged) | 18%  (-4%) | 48%  (unchanged) | *ns* |

Table SA 4. Cause of COPD admissions. Only five leading causes are mentioned.

| ***Cause of admission*** | ***2007*** | ***2008*** | ***2009*** | ***2010*** | ***2011*** | ***2012*** | ***2007 – 2012***  ***% change*** | ***p-value for monotonic trend**** |
| --- | --- | --- | --- | --- | --- | --- | --- | --- |
| **Cardiac failure (%)** | 10,12 | 10,66 | 11,05 | 12,16 | 12,04 | 12,33 | +21,91% | *.024* |
| ***Related mortality (%)*** | *9.63* | *10.20* | *10.09* | *10.24* | *10.16* | *10.75* | *+11.6%* | *.133* |
| **Pneumonia (%)** | 18,80 | 19,59 | 20,06 | 19,27 | 19,72 | 17,09 | -9,12% | *.999* |
| ***Related mortality(%)*** | *6.99* | *7.08* | *7.69* | *8.39* | *8.35* | *9.59* | *+37.2%* | *.024* |
| **Pneumothorax (%)** | 1,01 | 1,03 | 1,04 | 1,10 | 1,03 | 1,06 | +4,77% | *.181* |
| ***Related mortality(%)*** | *2.05* | *1.56* | *1.94* | *2.69* | *2.64* | *2.40* | *+17.1%* | *.452* |
| **Upper Airway infection (%)** | 46,51 | 46,76 | 47,51 | 48,36 | 50,89 | 54,49 | +17,16% | *.009* |
| ***Related Mortality(%)*** | *7.19* | *7.17* | *7.41* | *7.19* | *7.00* | *7.13* | *-0.88%* | *.260* |
| **Pulmonary embolism (%)** | 2,57 | 2,57 | 2,50 | 2,71 | 2,54 | 2,65 | +3,08% | *.848* |
| ***Related Mortality(%)*** | *8.44* | *7.53* | *7.13* | *7.31* | *7.31* | *6.44* | *-23.69%* | *.133* |

**p-*value for monotonic trend= Mann Kendall *p-*values are reported.

Table SA 5. Top seven medical acts performed during hospitalizations for COPD exacerbations. 2007 and 2012 data and variation are presented.

| **Medical Act** | **Year** | **Total** | **Category 4**  **(ICU-MV)** | **Category 3**  **(ICU-No MV)** | **Category 2 (ward-sign of ARF)** | **Category 1 (ward-No ARF)** |
| --- | --- | --- | --- | --- | --- | --- |
|  |  |  |  |  |  |  |
| **Electrocardiogram**  **N**  **(N/stay)** | 2007 | 81490  (0.70) | 14484  (1.41) | 6392  (1.24) | 16525  (0.66) | 44089  (0.59) |
|  | 2012 | 97740  (0.73) | 20663  (1.42) | 7109  (1.15) | 17087  (0.71) | 52881  (0.60) |
|  | variation per stay, *p*-value | +3.9%  *p*<.001 | +0.6% | -6.9% | +8.4% | +1.3% |
| **Chest X-ray**  **N**  **(N/stay)** | 2007 | 156372  (1.35) | 16196  (1.58) | 7679  (1.49) | 35411  (1.40) | 97086  (1.29) |
|  | 2012 | 108140  (0.81) | 14077  (0.97) | 5851  (0.95) | 20085  (0.83) | 68127  (0.77) |
|  | variation per stay, *p*-value | -40.1%  *p*<.001 | -38.7% | -36.2% | -40.5% | -40.7% |
| **Arterial blood**  **gases**  **N**  **(N/stay)** | 2007 | 41133  (0.36) | 4688  (0.46) | 1883  (0.37) | 10550  (0.42) | 24012  (0.32) |
|  | 2012 | 44896  (0.34) | 5924  (0.41) | 2085  (0.34) | 9743  (0.41) | 27144  (0.31) |
|  | variation per stay, *p*-value | -5.5%  *p*<.001 | -10.9% | -7.3% | -3.2% | -4.5% |
| **Trans-thoracic**  **echocardiogram**  **N**  **(N/stay)** | 2007 | 25158  (0.22) | 5134  (0.50) | 2566  (0.50) | 4343  (0.17) | 13115  (0.17) |
|  | 2012 | 31654  (0.24) | 7408  (0.51) | 3517  (0.57) | 4337  (0.18) | 16392  (0.18) |
|  | variation per stay, *p*-value | +9.0%  *p*<.001 | +1.8% | +14.7% | +4.7% | +5.6% |
| **Spirometry**  **N**  **(N/stay)** | 2007 | 93859  (0.81) | 8526  (0.83) | 3655  (0.70) | 22818  (0.90) | 58860  (0.79) |
|  | 2012 | 93554  (0.70) | 10783  (0.74) | 3867  (0.63) | 18743  (0.78) | 60161  (0.68) |
|  | variation per stay, *p*-value | -13.7%  *p*<.001 | -10.8% | -11.5% | -13.9% | -13.7% |
| **Thoracic**  **CT-scan**  **N**  **(N/stay)** | 2007 | 20311  (0.18) | 2857  (0.28) | 1181  (0.23) | 4143  (0.16) | 12130  (0.16) |
|  | 2012 | 31529  (0.24) | 5214  (0.36) | 1907  (0.31) | 5371  (0.22) | 19037  (0.21) |
|  | variation per stay, *p*-value | +34.4%  *p*<.001 | +28.7% | +35.1% | +36.0% | +32.6% |
| **Flexible**  **bronchoscopy**  **N**  **(N/stay)** | 2007 | 16443  (0.14) | 5636  (0.55) | 704  (0.14) | 2650  (0.11) | 7453  (0.10) |
|  | 2012 | 14693  (0.11) | 5688  (0.39) | 519  (0.08) | 1983  (0.08) | 6503  (0.07) |
|  | variation per stay, p-value | -22.6%  *p*<.001 | -28.8% | -38.3% | -21.5% | -26.3% |
